# Factors influencing job satisfaction and turnover intention among nurses in the UAE

**DOI:** 10.1371/journal.pone.0344434

**Published:** 2026-03-12

**Authors:** Mohammed Al Maqbali, Muna Al Shehhi, Fatima Al Hosani, Ciara Hughes

**Affiliations:** 1 Department of Nursing, Fatima College of Health Sciences, Al Ain, United Arab Emirates; 2 Department of Nursing, Sheikh Shakhbout Medical City, Abu Dhabi Health Services Company (SEHA), Abu Dhabi, United Arab Emirates; 3 Al Marfa Hospital, Abu Dhabi Health Services Company (SEHA), Abu Dhabi, United Arab Emirates; 4 Institute of Nursing and Health Research, School of Health Sciences, Ulster University, Belfast, Northern Ireland, United Kingdom; Prince Sattam bin Abdulaziz University, SAUDI ARABIA

## Abstract

Job satisfaction and turnover intention are critical factors influencing nurse retention and healthcare outcomes. This study explores the impact of organizational policies, work environment, patient care quality, and individual factors on job satisfaction and turnover intention among nurses in the UAE. A cross-sectional survey design was employed, with data collected between August and November 2024 from 313 nurses working across various healthcare settings in the UAE. Validated self-reported questionnaires, including the Job Satisfaction Scale and a Turnover Intention Measure, were used. The survey assessed job satisfaction, turnover intention, and their associations with key organizational and individual factors. Data were analysed using descriptive statistics, chi-square tests, and logistic regression to identify significant relationships and prevalence rates. The findings showed that 73.5% of nurses were satisfied with their jobs, while 20.1% reported an intention to leave. Job satisfaction was positively associated with supportive organizational policies, a positive work environment, higher patient care quality, and individual characteristics. Turnover intention was lower compared to global benchmarks, suggesting the potential impact of favourable workplace practices and systems in the UAE. Enhancing job satisfaction and reducing turnover intention require supportive policies, positive work environments, and access to professional development opportunities.

## 1 Introduction

Nurses are essential to healthcare, directly impacting patient outcomes through their work. However, the profession faces challenges like high stress, demanding environments, and critical decision-making responsibilities [1,2]. These issues, influenced by organizational policies, individual traits, and work conditions, affect job satisfaction and turnover intention [3,4]. Nursing combines compassionate care with technical expertise, requiring nurses to manage complex patient needs, collaborate with healthcare teams, and adapt to evolving practices. Although rewarding, these roles can lead to significant stress, affecting well-being and retention. Understanding these factors is crucial for fostering nurse satisfaction and retention [5,6].

In the United Arab Emirates (UAE), rapid healthcare advancements align with the country’s socioeconomic growth. Nurses play a key role but face challenges such as heavy workloads, high stress levels, and cultural diversity within a multicultural workforce. The nursing workforce in the UAE is uniquely characterized by its heavy reliance on expatriate professionals, who make up the vast majority of nurses in both the public and private sectors [7]. This structural dependence on internationally recruited staff presents distinct challenges related to labor mobility, job security, and retention [8]. In addition, multicultural team dynamics shaped by linguistic, cultural, and professional diversity demand adaptive communication, effective collaboration, and strong leadership skills, all of which can significantly influence job satisfaction and workplace integration [9].

Frequent staff turnover, often driven by contractual limitations and regional job migration trends, further complicates workforce stability. These contextual factors must be explicitly addressed to understand the challenges faced by nurses in the UAE and to inform retention strategies grounded in local realities. Addressing the factors that influence job satisfaction and turnover intention is essential to meet the unique needs of nurses in the UAE.

Turnover intention, defined as the likelihood that a nurse plans to leave their current position within a specific period, is a critical concern in the nursing profession. It negatively affects patient safety, care quality, and organizational performance [10]. High turnover rates are associated with increased workloads for remaining staff, reduced morale, and higher recruitment and training costs [11]. Job satisfaction is widely recognized as a key determinant of nurse retention and is influenced by factors such as effective leadership, fair compensation, opportunities for promotion, recognition of effort, and supportive interpersonal relationships [12,13]. In contrast, dissatisfaction frequently arises from excessive patient loads, inflexible scheduling, limited participation in decision-making, poor supervisory support, and a lack of access to professional development resources [14]. These conditions contribute to emotional exhaustion and burnout, which are closely linked to an increased intention to leave the profession or seek employment elsewhere.

Organizational policies play a critical role in shaping nurse satisfaction. Policies that promote well-being, career development, and work-life balance enhance retention, while rigid schedules, inadequate staffing, and limited growth opportunities increase turnover intention [15]. Work environment factors like team collaboration, job security, and resource availability are also pivotal [16]. Collaborative teams improve satisfaction by fostering shared responsibility, while safe, well-resourced environments support effective care delivery. However, resource limitations and job insecurity contribute to dissatisfaction [17].

This study explores the factors influencing job satisfaction and turnover intention among nurses in the UAE, focusing on organizational policies, individual characteristics, and work environment factors. Through examining these relationships, the study aims to inform evidence-based strategies to improve nurse retention, enhance professional well-being, and support healthcare system sustainability in the UAE.

## 2 Methodology

### 2.1 Study design

A cross-sectional study design was employed in accordance with the STROBE (Strengthening the Reporting of Observational Studies in Epidemiology) guidelines [18].

### 2.2 Sample

The target population included registered nurses working in public and private healthcare facilities across various regions of the UAE, with an estimated total population of approximately 12,000 nurses at the time of data collection. A minimum sample size of 300 was calculated using the Raosoft online sample size calculator [19], based on a 95% confidence level, a 5% margin of error, and an assumed response distribution of 50%. These assumptions were selected following Raosoft’s recommended parameters for large populations when the true population proportion is unknown and to account for expected non-response.

Nurses were recruited using a convenience sampling method. Invitations were distributed electronically via professional networks, hospital nursing departments, and online platforms. Only registered nurses actively working in the UAE were included in the study.

### 2.3 Data collection

A link to the online survey was distributed via social media platforms, primarily WhatsApp and email, targeting nurses across various healthcare settings in the UAE. The survey link was accompanied by an information sheet outlining the study’s purpose, confidentiality assurances, and the voluntary nature of participation. Data collection took place over a four-month period, from August 1 to November 30, 2024.

Reminder messages were sent every two weeks, totalling eight, to reach nurses across different hospital units. To prevent duplicate responses, the survey was set to allow only one submission per device, and participants were instructed to complete it only once.

### 2.4 Instrument

The survey consisted of four sections: demographics, demographic variables, Turnover Intention Scale (TIS-3), Job Satisfaction Scale, and Comprehensive Nursing Work Environment Scale.

#### 2.4.1 Demographics.

Information about participants’ age, sex, marital status, level of education, year of working, setting, and comorbidities were collected.

#### 2.4.2 Turnover intention scale.

Turnover intention was measured using the original English version of the 3-item Turnover Intention Scale (TIS-3) [20]. Each item was rated on a 5-point Likert scale (1 = never, 5 = very often). Higher scores indicated a greater intention to leave. A score ≥3.0 reflected an intention to leave, while <3.0 indicated no intention [21].

The TIS-3 has demonstrated acceptable reliability and validity in previous research. It has a Cronbach’s α of 0.77, indicating good internal consistency, and a Content Validity Index of 0.68. In the current study, the scale demonstrated excellent internal consistency and reliability, with a Cronbach’s alpha (α) of 0.86.

#### 2.4.3 Job satisfaction.

Job satisfaction was assessed using the original English version of a validated 4-item scale [22], rated on a 5-point Likert scale (1 = strongly disagree, 5 = strongly agree). Higher mean scores indicated greater job satisfaction (≥3.0 = satisfied, < 3.0 = dissatisfied). The scale has demonstrated strong psychometric properties, with a Cronbach’s alpha of 0.85, indicating excellent internal consistency [23]. In the present study, the scale showed good reliability with a Cronbach’s alpha of 0.80. Although concise, this scale has been widely used in previous nursing and organizational studies as a reliable measure of overall job satisfaction, offering a practical and psychometrically robust option for large survey-based research [23–25].

#### 2.4.4 Comprehensive nursing work environment.

The Comprehensive Nursing Work Environment was assessed using a modified English version of the Practice Environment Scale of the Nursing Work Index (PES-NWI), originally developed by [26]. The modified version measured four dimensions: organizational policies (6 items), individual characteristics (6 items), work environment (6 items), and patient care quality (10 items). Each item was rated on a 5-point Likert scale (1 = strongly disagree, 5 = strongly agree). Scores above 3 indicated a favourable environment. Cronbach’s alpha for the total scale was 0.91, demonstrating excellent reliability. In this study, the scale demonstrated strong internal consistency, with the following Cronbach’s alpha values reported for each dimension: Organizational Policies (α = 0.76), Individual Characteristics (α = 0.76), Work Environment (α = 0.67), and Patient Care Quality (α = 0.86). The total scale reliability was excellent, with a Cronbach’s alpha of 0.91. The modifications were limited to item rewording and cultural adaptation to ensure contextual relevance to the UAE healthcare setting, and the revised version was reviewed by nursing research experts to maintain content validity.

#### 2.4.5 Data analysis.

The data were exported to the Statistical Package for Social Science (SPSS) version 25 for analysis. Descriptive statistics, including means, standard deviations, frequencies, and percentages, were calculated for all variables. Chi-square tests were performed to examine associations between categorical variables and job satisfaction or turnover intention. Independent t-tests and One-way Analysis of Variance (ANOVA) was used to compare mean differences across demographic variables. Logistic regression analysis was conducted to identify the predictors of job satisfaction and turnover intention, focusing on organizational policies, individual characteristics, work environment, and patient care quality. A p-value of <0.05 was considered statistically significant. Given the exploratory nature of this study, no formal adjustment for multiple comparisons (e.g., Bonferroni correction) was applied; however, findings were interpreted with caution to minimize the risk of Type I error.

#### 2.4.6 Ethical considerations.

The study was conducted under the approval of the Abu Dhabi Health Research and Technology Ethics Committee (HREC SEHA-IRB-683) and adhered to the Helsinki Ethical Guidelines. All ethical requirements were met. Participant confidentiality and privacy were maintained by ensuring anonymity throughout the study. The participant information sheet and consent form were displayed on the first screen of the survey tool. Informed consent was obtained electronically, with participants indicating their agreement to participate by clicking a confirmation button. Those who did not provide consent were automatically redirected to the end of the survey, thereby ensuring voluntary participation. The consent process described here was fully aligned with the procedures submitted to and approved by the ethics committee.

## 3 Result

A total of 313 nurses participated in the study. The majority were female (252, 80.5%) and aged 31–40 years (166, 53%). Most participants (263, 84%) were married. The majority held a Bachelor’s degree (232, 74.1%) and worked in hospitals (283, 90.4%). Most participants had 11–15 years of experience (108, 34.5%). Among participants, 73.5% (230) were satisfied with their jobs, while 26.5% (83) were dissatisfied. Regarding turnover intention, 20.1% (63) reported an intention to leave, while 79.9% (250) had no intention to leave (Table 1).

**Table 1:** Demographic characteristic of participants with job satisfaction and intention to leave category (N = 313).

			Satisfied n = 230 (73.5%)		Dissatisfied n = 83(26.5%)			Intention to Leave n = 63(20.1%)		No Intention to Leave n = 250 (79.9%)		
	n	%	n	%	n	%	*p*	n	%	n	%	*p*
**Gender**							.95					.79
Male	61	19.5	45	44.5	16	19.3		13	20.6	48	19.2	
Female	252	80.5	185	80.4	67	66.8		50	79.4	202	80.8	
**Age**							.71					.12
21-30	27	8.6	20	8.7	7	8.40		5	7.9	22	8.8	
31-40	166	53	121	52.6	45	54.2		29	46.	137	54.8	
41-50	83	26.5	59	25.7	24	28.9		24	38.1	59	23.6	
Over 51	37	11.8	30	13	7	8.4		5	7.9	32	12.8	
**Marital Status**							.79					.23
Married	263	84	194	84.3	69	83.1		56	88.9	207	82.8	
Single	50	16	36	15.7	14	16.9		7	11.1	43	17.2	
**Education Level**							.16					.01
Diploma	28	8.9	23	10	5	6		0	0	28	11.2	
Bachelor	232	74.1	164	71.3	68	81.9		50	79.4	182	72.8	
Master Or PhD	53	16.9	43	18.7	10	12.		13	20.6	40	16	
**Working In**							.08					.62
Hospitals	283	90.4	212	92.2	71	85.5		58	92.1	225	90	
Primary Health Care	30	9.6	18	7.8	12	14.5		5	7.9	25	10	
**Working Experience**							.05					.001
Less Than 2	12	3.8	9	3.9	3	3.6		1	1.6	11	4.4	
3-5	27	8.6	19	8.3	8	9.6		5	7.9	22	8.8	
6-10	49	15.7	40	17.4	9	10.8		2	3.2	47	18.8	
11-15	108	34.5	69	30	39	47		35	55.6	73	29.2	
16-20	59	18.8	50	21.7	9	10.8		10	15.9	49	19.6	
More than 20	58	18.5	43	18.7	15	18.1		10	15.9	48	19.2	
**Comorbidities**							.98					.79
Yes	38	12.1	28	12.2	10	12		7	11.1	31	12.4	
No	275	87.9	202	87.8	73	88		56	88.9	219	87.6	
	**M**	**SD**	**M**	**SD**	**M**	**SD**	***P***	**M**	**SD**	**M**	**SD**	***P***
**Organizational Policies**	3.18	0.70	3.33	0.61	2.78	0.78	<.001	2.86	0.62	3.27	0.69	<.001
**Individual**	3.30	0.65	3.46	0.58	2.84	0.59	<.001	2.94	0.50	3.39	0.65	<.001
**Work Environment**	3.34	0.52	3.47	0.46	2.96	0.51	<.001	3.05	0.43	3.41	0.52	<.001
**Patient Care Quality**	3.88	0.53	4.01	0.43	3.51	0.60	<.001	3.66	0.53	3.93	0.52	<.001

**Fig 1:**
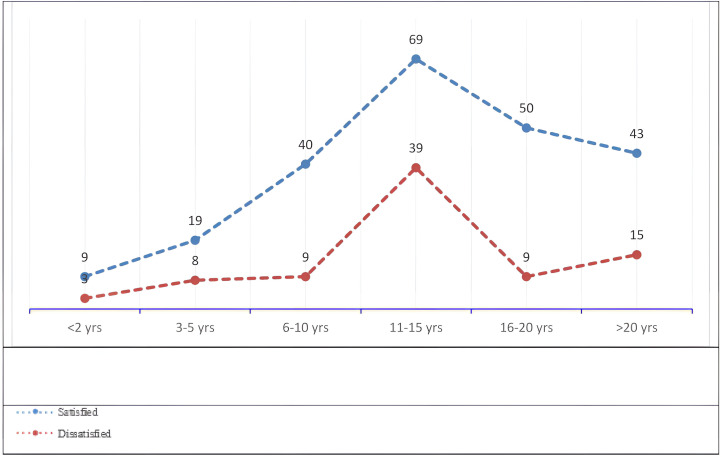
Distribution of job satisfaction levels among nurses according to years of clinical experience.

Significant differences were found in job satisfaction based on years of experience. Nurses with 11–15 years of experience reported the highest satisfaction (69, 30%), while those with fewer than 10 years of experience reported lower satisfaction (49, 21.3%) (Fig 1). Nurses with more than 20 years of experience (43, 18.7%) were less likely to be satisfied compared to those with 16–20 years (50, 21.7%). Participants with higher satisfaction had significantly higher scores for organizational policies (3.33 vs. 2.78; p < .001), individual factors (3.46 vs. 2.84; p < .001), work environment (3.47 vs. 2.96; p < .001), and patient care quality (4.01 vs. 3.51; p < .001).

**Fig 2:**
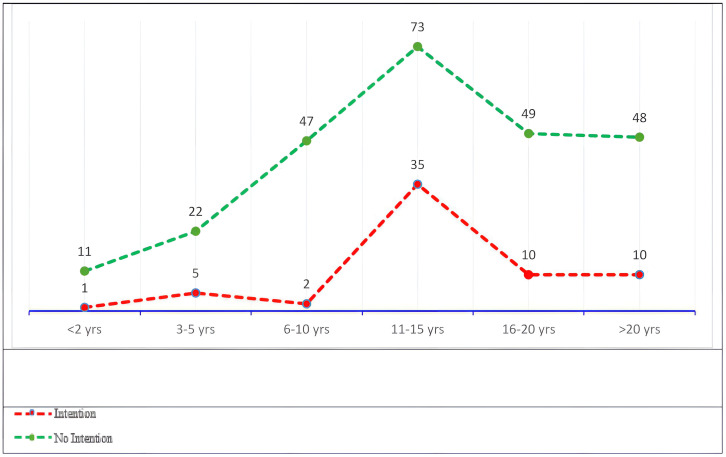
Distribution of turnover intention among nurses according to years of clinical experience.

There were significant differences in turnover intention based on education level and work experience (Table 1). Participants with a Diploma (5, 6%) reported a higher intention to leave compared to those with a Bachelor’s degree (50, 21.6%) or higher (13, 20.6%) (p = 0.01). Additionally, nurses with 11–15 years of experience (35, 55.6%) had a significantly higher intention to leave compared to those with fewer (1–10 years: 8, 12.7%) or more years of experience (≥16 years: 20, 31.7%) (p = 0.001) (Fig 2). Significant differences were found in turnover intention between participants with and without intention to leave for organizational policies (2.86 vs. 3.27; p < .001), individual factors (2.94 vs. 3.39; p < .001), work environment (3.05 vs. 3.41; p < .001), and patient care quality (3.66 vs. 3.93; p < .001).

### 3.1 Correlations

Significant correlations were found between job satisfaction and organizational policies (r = 0.583, p < 0.01), individual factors (r = 0.633, p < 0.01), work environment (r = 0.560, p < 0.01), and patient care quality (r = 0.525, p < 0.01). Turnover intention showed significant negative correlations with organizational policies (r = −0.480, p < 0.01), individual factors (r = −0.504, p < 0.01), work environment (r = −0.442, p < 0.01), and patient care quality (r = −0.347, p < 0.01) (seen Table 2). Although all associations were statistically significant, the moderate correlation strengths suggest that these relationships, while meaningful, should be interpreted in terms of both practical and contextual relevance rather than statistical significance alone.

**Table 2:** Correlations among job satisfaction, intention to leave, organizational factors, individual factors, work environment, and patient care quality.

		α^@^	1	2	3	4	5	6	7
**1**	Job Satisfaction	.80	1						
**2**	Intention to Leave	.86	−.573**	1					
**3**	Organizational	.76	.583**	−.480**	1				
**4**	Individual	.76	.633**	−.504**	.773**	1			
**5**	Work Environment	.67	.560**	−.442**	.656**	.664**	1		
**6**	Patient Care Quality	.86	.525**	−.347**	.524**	.542**	.634**	1	
**7**	Total	.91	.675**	−.514**	.859**	.863**	.845**	.829**	1

** Correlation is significant at the 0.01 level (2-tailed).

@ **α =** Cronbach’s alpha.

### 3.2 Predictors of job satisfaction

All variables were included in the logistic regression model to identify predictors of job satisfaction. The model was statistically significant (χ2 = 114.33, p < 0.001), explaining between 30.6% (Cox & Snell R^2^) and 44.6% (Nagelkerke R^2^) of the variance, with an overall prediction accuracy of 84%. Among the predictors, individual factors (OR=2.751, 95% CI = 1.179–6.422, p = 0.019) and patient care quality (OR=3.666, 95% CI = 1.574–8.541, p = 0.003) were found to be significant. Nurses with higher scores in individual factors and patient care quality were more likely to report job satisfaction (Table 3).

**Table 3:** Logistic regression results for predictors of job satisfaction odds ratio (95% CI).

	Total	Satisfied n = 230 (73.5%)	Dissatisfied n = 83(26.5%)	OR (95%CI)	*P*
	n (%)	n (%)	n (%)		
**Gender**					
Male	61(19.5)	45(44.5)	16(19.3)	**Reference**	
Female	252(80.5)	185(80.4)	67(66.8)	0.872(0.386-1.969)	0.74
**Age**					
21-30	27(8.6)	20(8.7)	7(8.4)	**Reference**	
31-40	166(53)	121(52.6)	45(54.2)	0.927(0.221-3.895)	0.92
41-50	83(26.5)	59(25.7)	24(28.9)	0.373(0.139-3.9)	0.72
Over 51	37(11.8)	30(13)	7(8.4)	0.824(0.122-5.549)	0.84
**Marital Status**					
Married	263(84)	194(84.3)	69(83.1)	**Reference**	
Single	50(16)	36(15.7)	14(16.9)	0.729(0.284-1.868)	0.51
**Education Level**					
Diploma	28(8.9)	23(10)	5(6)	**Reference**	
Bachelor	232(74.1)	164(71.3)	68(81.9)	0.768(0.2-2.951)	0.70
Master Or PhD	53(16.9)	43(18.7)	10(12)	1.697(0.359-8.025)	0.51
**Working In**					
Hospitals	283(90.4)	212(92.2)	71(85.5)	**Reference**	
Primary Health Care	30(9.6)	18(7.8)	12(14.5)	0.668(0.22-2.028)	0.48
**Working Experience**					
Less Than 2	12(3.8)	9(3.9)	3(3.6)	**Reference**	
3-5	27(8.6)	19(8.3)	8(9.6)	1.477(0.162-13.443)	0.73
6-10	49(15.7)	40(17.4)	9(10.8)	0.794(0.099-6.339)	0.83
11-15	108(34.5)	69(30)	39(47)	0.507(0.062-4.112)	0.53
16-20	59(18.8)	50(21.7)	9(10.8)	1.56(0.165-14.789)	0.70
More than 20	58(18.5)	43(18.7)	15(18.1)	1.088(0.117-10.148)	0.94
**Comorbidities**					
Yes	38(12.1)	28(12.2)	10(12)	**Reference**	
No	275(87.9)	202(87.8)	73(88)	0.667(0.241-1.848)	0.44
No Intention to Leave	250(79.9)	46(55.4)	204(88.7)	**Reference**	
Intention to Leave	63(20.1)	26(11.3)	37(44.6)	0.251(0.118-0.536)	<.001
	**Mean (SD)**	**Mean (SD)**	**Mean (SD)**		
Organizational Policies Factors	3.18(0.7)	3.33(0.61)	2.78(0.78)	0.761(0.36-1.61)	0.48
Individual Factors	3.3(0.65)	3.46(0.58)	2.84(0.59)	2.751(1.179-6.422)	0.02
Work Environment Factors	3.34(0.52)	3.47(0.46)	2.96(0.51)	2.639(0.912-7.633)	0.07
Patient Care Quality Factors	3.88(0.53)	4.01(0.43)	3.51(0.6)	3.666(1.574-8.541)	0.00

**a Variable(s) entered on step 1:** Gender, Age, Marital Status, Education Level, working in, Years of Working, Intention to Leave Category Cat, Organizational Factors, Individual Factors, Work Environment Factors, Patient Care Quality Factors.

### 3.3 Predictors of turnover intention

All variables were entered into a logistic regression model to identify predictors of turnover intention. The model was statistically significant (χ² = 78.875, p < .001), explaining 24% to 37% of the variance (Cox & Snell R^2^ and Nagelkerke R^2^, respectively), with a prediction accuracy of 79%. Four variables were significant predictors. Job satisfaction was negatively associated with turnover intention (OR = 0.272, 95% CI = 0.127–0.581, p < .001), while nurses with 11–15 years of experience were more likely to report turnover intention (OR = 24.384, 95% CI = 1.373–432.975, p = .03); however, the wide confidence interval indicates potential estimate instability. Nurses aged 31–40 years were less likely to report turnover intention than those aged 21–30 (OR = 0.12, 95% CI = 0.019–0.76, p = .02). Work environment perceptions were marginally associated with lower turnover intention (OR = 0.355, 95% CI = 0.124–1.015, p = .05). The diploma group was excluded due to complete separation. Regression results are detailed in Table 4. While several predictors were statistically significant, the wide confidence intervals for some estimates indicate that practical implications should be interpreted with caution, and further studies with larger, more balanced samples are needed to confirm these associations.

**Table 4:** Logistic regression results for predictors of intention to leave odds ratio (95% CI).

	Total	Intention to Leave n = 63(20.1%)	No Intention to Leave n = 250 (79.9%)	OR (95%CI)	*P*
	n (%)	n (%)	n (%)		
**Gender**					
Male	61(19.5)	13(20.6)	48(19.2)	**Reference**	
Female	252(80.5)	50(79.4)	202(80.8)	0.957(0.417-2.198)	0.92
**Age**					
21-30	27(8.6)	5(7.9)	22(8.8)	**Reference**	
31-40	166(53)	29(46)	137(54.8)	0.12(0.019-0.76)	0.02
41-50	83(26.5)	24(38.1)	59(23.6)	0.515(0.072-3.663)	0.51
Over 51	37(11.8)	5(7.9)	32(12.8)	0.356(0.04-3.134)	0.35
**Marital Status**					
Single	50(16)	7(11.1)	43(17.2)	**Reference**	
Married	263(84)	56(88.9)	207(82.8)	1.859(0.612-5.646)	0.27
**Education Level**					
Diploma	28(8.9)	0(0)	28(11.2)	Removed *	
Bachelor	232(74.1)	50(79.4)	182(72.8)	**Reference**	
Master Or PhD	53(16.9)	13(20.6)	40(16)	1.513(0.643-3.563)	0.34
**Working In**					
Hospitals	283(90.4)	58(92.1)	225(90)	**Reference**	
Primary Health Care	30(9.6)	5(7.9)	25(10)	0.572(0.153-2.142)	0.41
**Working Experience**					
Less Than 2	12(3.8)	1(1.6)	11(4.4)	**Reference**	
3-5	27(8.6)	5(7.9)	22(8.8)	4.279(0.298-61.531)	0.29
6-10	49(15.7)	2(3.2)	47(18.8)	1.22(0.066-22.455)	0.89
11-15	108(34.5)	35(55.6)	73(29.2)	24.384(1.373-432.975)	0.03
16-20	59(18.8)	10(15.9)	49(19.6)	8.319(0.427-161.973)	0.16
More than 20	58(18.5)	10(15.9)	48(19.2)	4.942(0.266-91.853)	0.28
**Comorbidities**					
Yes	38(12.1)	7(11.1)	31(12.4)	**Reference**	
No	275(87.9)	56(88.9)	219(87.6)	1.863(0.638-5.439)	0.26
Dissatisfied	83(26.5)	37(44.6)	46(55.4)	**Reference**	
Satisfied	230(73.5)	26(11.3)	204(88.7)	0.272(0.127-0.581)	<.001
	**Mean (SD)**	**Mean (SD)**	**Mean (SD)**		
Organizational Policies Factors	3.18(0.7)	2.86(0.62)	3.27(0.69)	1.005(0.458-2.209)	0.99
Individual Factors	3.3(0.65)	2.94(0.5)	3.39(0.65)	0.688(0.291-1.628)	0.40
Work Environment Factors	3.34(0.52)	3.05(0.43)	3.41(0.52)	0.355(0.124-1.015)	0.05
Patient Care Quality Factors	3.88(0.53)	3.66(0.53)	3.93(0.52)	1.037(0.445-2.42)	0.93

**a Variable(s) entered on step 1**: Gender, Age, Marital Status, Education Level, Working in, Years of Working, Total Organizational Factors, Total Individual Factors, Total Work Environment Factors, Total Patient Care Quality Factors, Job Satisfaction Category.

* Due to complete separation in the Diploma group (0 outcome cases), this category was excluded from the regression model to ensure model stability.

## 4 Discussion

The aim of this study was to investigate whether organizational policies, individual factors, work environment, and patient care quality influence job satisfaction and turnover intention among nurses in the UAE, and how these relationships manifest. The study found that these factors were significantly associated with job satisfaction, with supportive organizational policies and a positive work environment playing critical roles in promoting higher satisfaction levels. Furthermore, the results demonstrated that higher satisfaction in these areas could predict reduced turnover intention, emphasizing the importance of fostering conducive working conditions. Overall, these findings provide a deeper understanding of the interplay between organizational and individual factors and highlight the potential of targeted interventions in enhancing job satisfaction and retention among nurses.

The findings revealed that 73.5% of participants were satisfied with their jobs, while 26.5% reported dissatisfaction, indicating a moderate level of job satisfaction that aligns with previous studies emphasizing the need for continued improvement [27]. Comparatively, this percentage of job dissatisfaction is lower than the findings of a meta-analysis of 278 studies, which reported a job dissatisfaction rate of 51% among nurses (Al Maqbali, 2024). This lower rate of dissatisfaction may be attributed to supportive workplace policies, including better staffing ratios, professional development opportunities, or recognition programs that help create a more positive work environment for nurses.

Notably, nurses with 11–15 years of experience reported the highest levels of job satisfaction. This may be attributed to a period of professional stability and mastery of skills, which likely enhances confidence and job fulfilment. Conversely, nurses with fewer years of experience reported lower satisfaction, possibly due to adjustment challenges, workload stress, or lack of support [24,28]. Nurses with more than 20 years of experience showed a slight decline in satisfaction, which may reflect career stagnation or burnout from prolonged exposure to workplace demands [29,30]. Interestingly, turnover intention also peaked among nurses with 11–15 years of experience. This may reflect a phase in which mid-career nurses, despite feeling competent and satisfied in their roles, begin seeking career advancement, leadership positions, or better employment opportunities. Organizational stagnation, limited promotion pathways, or increased administrative responsibilities during this period may contribute to higher turnover intention despite moderate job satisfaction [31,32].

The present study found that 20.1% of nurses reported an intention to leave their current positions. This rate is broadly aligned with international findings and falls within the global range documented in the literature. A recent meta-analysis encompassing data from 17 countries, including the United States, Canada, China, and several European nations, reported turnover intention rates ranging from 2.2% to 50%, with a pooled global prevalence of 18% [33]. Similarly, another systematic review and meta-analysis involving 14 countries estimated a global turnover intention rate of 16% (95% CI: 14% to 17%) [34]. Although the rate observed in the current study is slightly higher than these pooled estimates, it remains well within the documented global variability. These findings emphasize the importance of contextualizing turnover intention within broader international trends and reinforce the need for targeted strategies to address organizational and systemic factors that influence nurse retention.

The study also revealed significant differences in turnover intention based on education level and years of experience. Nurses holding Diplomas exhibited the highest turnover intention, likely due to limited career advancement opportunities compared to those with Bachelor’s or higher degrees [33,35]. Additionally, turnover intention peaked among nurses with 11–15 years of experience, a finding that suggests mid-career challenges such as role fatigue, limited upward mobility, or dissatisfaction with organizational support [36].

Significant correlations were observed between job satisfaction and turnover intention. Nurses with higher scores for organizational policies, individual factors, work environment, and patient care quality were less likely to report turnover intention. This aligns with existing literature indicating that favourable working conditions and supportive organizational strategies are essential in retaining staff and improving job satisfaction [37,38].

Logistic regression results further emphasized the role of individual factors and patient care quality as significant predictors of job satisfaction. This finding highlights the importance of addressing nurses’ individual needs and maintaining high standards of patient care to promote professional fulfilment. Interestingly, job satisfaction and years of experience (11–15 years) were also identified as significant predictors of turnover intention. Although this may appear contradictory and suggest that mid-career nurses report both high satisfaction and a high intention to leave, it may reflect underlying issues such as career stagnation, unmet expectations, or limited opportunities for advancement [39,40]. Mid-career nurses may feel competent and fulfilled in their current roles yet simultaneously perceive a lack of professional growth or organizational support, which could prompt them to seek alternative opportunities [41]. These findings underscore the need for targeted retention strategies that address the specific concerns of experienced nurses and offer clear pathways for career progression.

The findings of this study emphasize the importance of enhancing job satisfaction among nurses through targeted organizational strategies. Job satisfaction can be improved through fostering supportive workplace policies, such as fair workload distribution, professional development opportunities, and recognition programs [42]. Additionally, improving nurse-patient ratios, providing sufficient resources, and creating a positive work environment can help reduce workplace stress and enhance satisfaction [8,43]. Leadership development is also crucial, as nurse managers play a pivotal role in promoting collaboration, communication, and motivation within their teams. Cultivating a supportive and inclusive culture ensures that nurses feel valued and fulfilled in their roles.

Addressing turnover intention is equally important, as it directly impacts the stability of the nursing workforce and patient care quality. The study’s relatively low turnover intention highlights the potential to further reduce this rate through flexible work schedules, career advancement opportunities, and regular engagement with staff to understand and address concerns [44,45]. Interventions focused on mental health support, resilience training, and stress management can also mitigate burnout and turnover intention [46]. Open communication channels between management and staff are essential for building trust and ensuring that nurses’ voices are heard. Addressing these factors not only reduces turnover intention but also creates a more stable and satisfied nursing workforce, ultimately benefiting patient outcomes.

In the context of the United Arab Emirates, these findings are particularly relevant to national workforce strategies, including the Emiratization policy, which aims to increase the participation of UAE nationals in the healthcare sector [47]. Retaining skilled and experienced nurses is critical to achieving sustainable workforce development under these initiatives. Furthermore, the UAE’s national strategy for nursing emphasizes the importance of improving work environments, expanding career pathways, and supporting professional development [47,48]. These areas are directly aligned with the factors identified in this study and are consistent with findings from other Middle Eastern countries, where similar workforce and retention challenges have been reported. Aligning retention strategies with these national goals can enhance workforce stability and contribute to improved healthcare outcomes across the country.

There are several limitations to this study that should be considered when interpreting the findings. First, the study employed a cross-sectional design, which limits the ability to establish causal relationships between job satisfaction, turnover intention, and the examined factors. Therefore, the observed associations should not be interpreted as evidence of causality, as the temporal sequence of events cannot be determined in this design. Longitudinal or experimental research designs are recommended for future studies to better understand the causality of these relationships. Second, the data were collected using self-reported measures, which may introduce response bias or inaccuracies due to social desirability or recall errors. Incorporating objective measures or triangulating data collection methods in future research could improve the reliability of the findings. Third, the use of convenience sampling through social media platforms such as WhatsApp may have introduced self-selection and selection biases, potentially affecting the representativeness of the sample and limiting the generalizability of the findings.

Fourth, the study was conducted among nurses in the UAE, which may limit the generalizability of the results to nurses in other countries or healthcare systems. Cultural and organizational differences in nursing practices and work environments should be taken into account when applying these findings to broader contexts. Fifth, no pilot testing was conducted within the UAE healthcare setting prior to data collection, which may be considered a minor limitation. Finally, while this study focused on individual and organizational factors influencing job satisfaction and turnover intention, it did not explore other potentially significant contributors, such as leadership styles, team dynamics, or patient care demands. Future research should consider a more comprehensive approach to identify additional factors that may influence nurses’ job satisfaction and retention. In addition, some regression models yielded wide confidence intervals, likely due to small subgroup sizes, which may indicate reduced precision of estimates. These results should therefore be interpreted with caution.

## 5 Conclusion

This study highlights the significant impact of organizational policies, work environment, and individual factors on nurses’ job satisfaction and turnover intention. The findings suggest that fostering supportive organizational policies, improving work conditions, and addressing sources of stress can enhance job satisfaction and reduce turnover intention. These insights provide valuable guidance for healthcare managers and policymakers in creating supportive work environments that promote nurse retention and improve patient care outcomes. Future research should explore additional factors to further strengthen strategies for enhancing satisfaction and reducing turnover.
